# The preparatory set: a novel approach to understanding stress, trauma, and the bodymind therapies

**DOI:** 10.3389/fnhum.2015.00178

**Published:** 2015-04-01

**Authors:** Peter Payne, Mardi A. Crane-Godreau

**Affiliations:** Microbiology and Immunology, Geisel School of Medicine at DartmouthLebanon, NH, USA

**Keywords:** somatic experiencing, preparatory set, body-oriented psychotherapy, stress, mind-body, trauma, post-traumatic stress disorder, meditative movement

## Abstract

Basic to all motile life is a differential approach/avoid response to perceived features of environment. The stages of response are initial reflexive noticing and orienting to the stimulus, preparation, and execution of response. Preparation involves a coordination of many aspects of the organism: muscle tone, posture, breathing, autonomic functions, motivational/emotional state, attentional orientation, and expectations. The organism organizes itself in relation to the challenge. We propose to call this the “preparatory set” (PS). We suggest that the concept of the PS can offer a more nuanced and flexible perspective on the stress response than do current theories. We also hypothesize that the mechanisms of body-mind therapeutic and educational systems (BTES) can be understood through the PS framework. We suggest that the BTES, including meditative movement, meditation, somatic education, and the body-oriented psychotherapies, are approaches that use interventions on the PS to remedy stress and trauma. We discuss how the PS can be adaptive or maladaptive, how BTES interventions may restore adaptive PS, and how these concepts offer a broader and more flexible view of the phenomena of stress and trauma. We offer supportive evidence for our hypotheses, and suggest directions for future research. We believe that the PS framework will point to ways of improving the management of stress and trauma, and that it will suggest directions of research into the mechanisms of action of BTES.

## Introduction

This paper is in response to two challenges: first, we offer an alternative perspective on the concept of stress, and second, we provide a clearer understanding of the mechanisms of action of body-mind therapeutic and educational systems (BTES). We propose the concept of the Preparatory Set (PS), defined as the unitary, largely subcortical, organization of the organism in preparation for response to environmental conditions. We address stress and BTES in the same discussion because we hypothesize that the maladaptive PS is the cause of stress, and that the effectiveness of BTES largely depends on addressing maladaptive PS. We suggest that these concepts offer a way of understanding the BTES, as well as a broader and more flexible way of understanding the phenomena referred to as “stress.”

### Stress

The term “stress” is widely used, and its significant contribution to human disease and suffering is well recognized (Kemeny and Schedlowski, 2007; Chida et al., 2008; McEwen, 2008; Fagundes et al., 2012; Van der Kolk et al., 2012; Everly and Lating, 2013). However, the term is often poorly defined. It has been used to refer to a subjective state, a physiological reaction, a neurochemical response, or the presence of a certain kind of external situation. For a review, see (Everly and Lating, 2013). Over 50 years after the first use of the term, there are enough questions left unresolved that we suggest that a different way of looking at these phenomena is needed. Below we list these key questions:

- What is the difference between “good” stress and “bad” stress?
- What distinguishes acute, chronic and traumatic stress, apart from the differing physiological responses?
- What is resilience to stress?
- What is appraisal, and how does it influence the stress response?
- What is the actual nature of stress, apart from its neurological and neurochemical correlates?

Simple physiological definitions of stress in terms of prolonged sympathetic arousal do not appear to answer these questions. We propose that the concept of the PS can clarify these issues.

### BTES

BTES, including meditation, meditative movement, Somatic education and body-oriented psychotherapies, offer approaches to alleviating human suffering that differ substantially from mainstream pharmacological, cognitive, exposure and exercise interventions (Everly and Lating, 2013). BTES address the person as an integrated whole, an approach quite different from the bio-medical approach of isolating, analyzing and treating the functioning of separate systems. BTES use movement, proprioception, interoception, posture, and various ways of attending to the body and bodily experience, rather than cognitive methods or conventional exercise approaches (Wright, 2000; Stuart, 2013; Kimmel et al., 2015). These factors have made it difficult for researchers to find appropriate conceptual frameworks for studying BTES (Kerr, 2002). As a result, despite substantial clinical and anecdotal evidence for efficacy, most BTES have not been well researched (with the exceptions of mindfulness meditation (Holzel et al., 2011a; Vago and Silbersweig, 2012; Tang and Posner, 2013) and Yoga (Roberts, 2013; Gard et al., 2014; Riley and Park, 2014).

## The preparatory set

### History

The term “preparatory set” has been in use in the scientific literature at least since 1918, in the context of studies of reaction time (Henmon, 1918). In subsequent decades the term was used in a number of contexts, and alternate names were used such as “organic set” (Young, 1925) and “quantitative set” (Bills and Brown, 1929). In 1941 Gibson critiqued the excessively broad use of the term (Gibson, 1941), and subsequent to that the term came to be used mainly in the context of the influence of cognitive expectation on perceptual and motor reaction time (Ruge et al., 2013).

Here we use the term to refer to the rapid, largely sub-cortical, preparation of the organism for response to the environment. We suggest that this preparation involves an organization of core features of the organism in readiness: physical posture and muscle tone, visceral state, affective or motivational state, arousal and orientation of attention, and (subcortical) cognitive expectations. This PS precedes, and influences, the complex human cortical responses of conscious appraisal and voluntary planning.

### Preparation and response

We suggest that an organism's response to the environment can be seen as having 3 phases:

- initial noticing and orienting to of the situation;
- preparation of response;
- execution of response (e.g., run away, run toward).

These phases do not necessarily follow a linear course; for instance, the execution of a response may be interrupted by a re-evaluation and preparation for an alternate response (Resulaj et al., 2009).

The initial orienting phase is unconscious, automatic and controlled by the brain stem reticular formation, especially the caudal areas which mediate generalized cortical and somatic arousal (Sokolov, 1963; Sarter et al., 2003). These rapid and automatic processes are not included in the preparatory response. Bull, in her attitude theory of emotion, also distinguishes between the preparatory response itself and the initial unconscious automatic reflex response (Bull, 1951).

Following this is the *preparation* for response. We suggest that this is a rapid, largely subcortical response; although it is not fully unconscious it is distinct from the conscious rational appraisal and voluntary decision-making process mediated by the cortical executive networks. It is an integrated readying of the whole organism to take action, and involves simultaneously posture, autonomic activity, affect, attention, and expectation. This phase is our principal focus.

The action phase, which follows, is the performance of the prepared response. It may happen almost immediately, after a delay, or not at all. Proprioceptive and exteroceptive feedback will inform the organism about the successful completion of the action (Gellhorn and Hyde, 1953; Suetterlin and Sayer, 2013), following which a new PS may form (See **Figure 2**).

### The five elements of the PS

**Figure 1:**
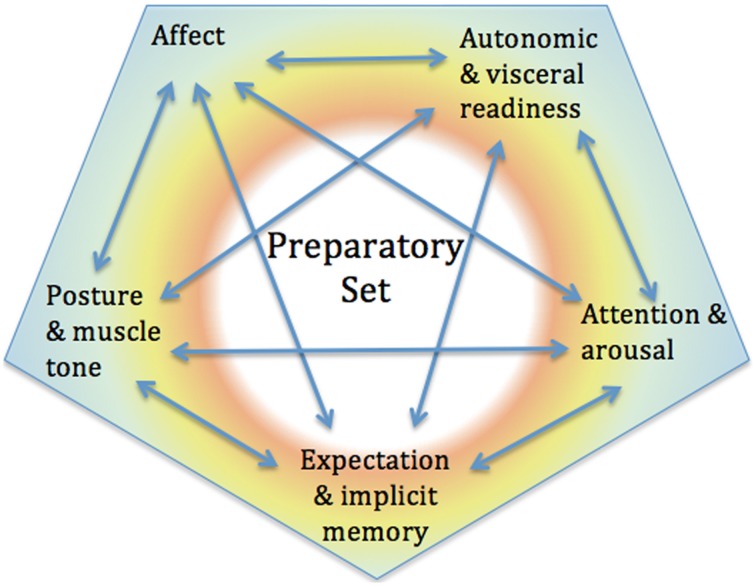
**The preparatory set and its five elements: affect, posture, and muscle tone, autonomic state, attention, and expectation all influence each other, forming a unitary response**.

The PS involves integrated action of the subcortical systems controlling muscle tone and posture, autonomic/visceral state, affect, attentional arousal, and expectation. It is a key point in our hypothesis that these all tend to respond together, as different facets of a single process. We hypothesize therefore that intervention in any one of these five aspects will tend to influence the others; for example, that changing posture may alter affective state, or changing the direction of attention may alter the autonomic state. We suggest that this hypothesis can explain the effects of the BTES, which use exactly these types of interventions. There is already significant evidence for strong mutual influence of many of these. Below we discuss each in turn, describing its function briefly and presenting evidence for its influence on other aspects of the PS (See Figure 1).

#### Posture, muscle tone, and breathing

Posture, muscle tone and breathing are closely interrelated. We choose here to discuss them together; to discuss them separately would add complexity to an already complex subject. The words “posture,” “attitude,” and “stance” can be used in multiple ways, as in: physical stance, emotional stance, cognitive stance. We believe this apparently metaphorical similarity points to the underlying reality of the intrinsic connection between posture, emotional attitude and cognitive attitude as hypothesized in the concept of PS.

##### Movement and posture

Preparation for movement involves the adoption of a posture. “Posture” here does not mean a completely static position, but a dynamic preparatory state involving small motions and changes in muscle tone. It may be distinguished from overt consummatory movements (such as running, reaching or eating). All behavior involves a continual shifting between preparatory and action phases.

Postural preparation underlies movement; and movement underlies life. Sperry has referred to the brain as a “motor brain” (Sperry, 1961). However the relation of motor function to affect and cognition remained relatively unexplored until the past decade (Downing, 2000). Proprioception refers to information coming to the brain about the position and movement of the body and is essential for coordinated movement (Sainburg et al., 1995; Riemann and Lephart, 2002). It comes principally from the muscle spindles and joint receptors, Pacinian corpuscles and free nerve endings in the connective tissue (Riemann and Lephart, 2002; van der Wal, 2009), and the vestibular apparatus. This information may be conscious or unconscious, and training can increase awareness of it (Hewett et al., 2002; Tsang and Hui-Chan, 2003). It has not received as much attention as interoception, but we suggest that it has an importance far beyond the mechanical coordination of the body.

##### Developmental aspects of posture

Posture and movement have been shown to be crucial for the early development of personality. Movement and the sense of movement are among the first abilities to develop in the infant (Thelen, 1995). Disturbances at this phase of development may profoundly damage later affective and cognitive development (Thelen, 1995). An infant's first communication is gestural/postural animation. This forms the basis for the later acquisition of language (Sheets-Johnstone, 2011; Esteve-Gibert and Prieto, 2014). Newborn infants imitate the bodily movements of adults (Meltzoff and Moore, 1983); infant development comes about largely through physical engagement of movement in relation to caregivers (Smith and Gasser, 2005). This suggests a central role for movement and movement preparation. Haselager (Haselager et al., 2011) conjectures that awareness of movement forms the basis for the development of the sense of self. The simulation of bodily experienced states and actions are significantly involved in memory (Ross et al., 2007), understanding (Barsalou et al., 2003; Pulvermüller, 2005), interpersonal communication (Hostetter and Alibali, 2004), social interaction (Sebanz et al., 2006), and spatial perception (Tversky, 2003). This supports the idea that postural and bodily aspects of the PS are intrinsically linked other aspects such as expectation, affect, and awareness.

##### Mechanical aspects of posture

Prior to an action “anticipatory postural adjustments” are made, anticipating the displacement of the body or body parts along a pathway toward a goal (Massion, 1992) and maintaining direction of attention and support of the center of gravity by the ground (Jung, 1981). Direction of attention and stable support are necessary for accurate organization of postural adjustments and controlled movement (Massion, 1994). These “reference cues” (Gurfinkel et al., 1981) integrate with internal representations of posture, especially the longitudinal axis of the body and its relation to gravity and support feedback (Macpherson et al., 1989; Mitteltaedt, 1989). We believe that emotional posture is closely linked to these mechanical aspects of posture (Weisfeld and Beresford, 1982; Bianchi-Berthouze et al., 2006). Postural preparation is functional, in that a certain goal is being prepared for (e.g., to attack or to run away). Some BTES work extensively with this aspect of posture. We suggest that conscious proprioceptive awareness of reflex postural preparedness may give rise to pleasant experiences of stability, readiness, and self-efficacy, or unpleasant feelings of lack of confidence and anxiety. See below for ways of testing this hypothesis.

Breathing likewise has a mechanical aspect, closely linked to posture in that efficient breathing and efficient posture support each other. It also has an autonomic and affective aspect. Voluntary control of breathing has been demonstrated to alter autonomic and affective state (Brown and Gerbarg, 2005a; Chan et al., 2010; Busch et al., 2012; Sano et al., 2015). Breath control is a key intervention in most BTES.

##### Emotional aspects of posture

Facial expression has long been recognized as an inherent aspect of emotion (Ekman et al., 1983; Levenson et al., 1990); engaging the smiling musculature produces positive affect (Strack et al., 1988). However the role of bodily postural expression has been less investigated. De Gelder (2006) proposes the term “emotional body language” (EBL) to refer to the characteristic postures and subtle movements of the body that express and communicate emotion. She suggests facial expression and bodily posture are two facets of a single process. Like facial expression, EBL is largely automatic rather than voluntary, and is controlled by a subcortical network involving the amygdala, pulvinar, striatum and superior colliculus; fully conscious experience of EBL is mediated by insula, somatosensory cortex, anterior cingulate and ventro-medial prefrontal cortex, and the perception of EBL in others by yet another network. In experiments involving the identification of emotion from photos, EBL was shown to be less ambiguous than facial expression, possibly because it shows the intended action more clearly (de Gelder and Hortensius, 2014). This supports our suggestion that intended action is a key aspect of affect as well as affective posture.

There is significant evidence for the connection between posture and emotional and cognitive states. The recently developed fields of embodied cognition (Varela et al., 1991; Wilson, 2002) and grounded cognition (Barsalou, 2008) offer many examples. Voluntary alteration of posture has been shown to influence risk behavior (Carney et al., 2010). An expansive posture induced a rise in testosterone, and a contracted posture induced a rise in cortisol (Carney et al., 2010). Smiling (a “posture” of the face) induced positive feelings and a slumped posture produced negative affect (Strack et al., 1988; Stepper and Strack, 1993). Thinking words related to pride alters posture (Oosterwijk et al., 2009); and a collapsed posture increases depressive thought (Weisfeld and Beresford, 1982).

Holstege's concept of the “emotional motor system” (EMS) (Holstege et al., 1996), supports these ideas. The EMS operates independently of the voluntary cortical control of movement, and is responsible for muscle tone and core body posture as well as expressive gesture. Holstege mentions that patients with pyramidal tract injury paralyzing the facial muscles can still smile in response to an emotional feeling (Holstege et al., 1996). Yawning, stretching and other reflexive function can occur even in the presence of extensive pyramidal lesions (Töpper et al., 2003). These findings support the existence of a subcortical connection between affective state and facial and bodily expression.

Panksepp, in considering the brain structures supportive of the core sense of self, regarded motor function as essential and definitional; he points to the periaqueductal gray (PAG) and surrounding midbrain regions as a prime candidate (Panksepp, 1998). The PAG also plays a central role in the EMS as hypothesized by Holstege (2013), and is centrally involved in the organization of integrated defensive and appetitive responses (Bandler et al., 1991). Panksepp suggests that at this basic level movement is inseparable from affect (Panksepp, 1998); we hypothesize these structures (PAG and adjacent midbrain nuclei, limbic region, and basal ganglia) form the principle neural substrate for the PS.

#### Autonomic response

The autonomic nervous system (ANS) readies the body for appropriate response to environmental challenge. It controls the activation of the cardio-respiratory system, the gastro-intestinal tract, sweat glands, hair follicles, and pupillary dilation via the sympathetic and parasympathetic nerves, as well as controlling aspects of the endocrine system by way of the pituitary. The ANS also strongly influences the motor system, controlling muscle tone via activation of the gamma efferent neurons, which influence the responsiveness of the muscles in preparation for complex defensive or appetitive movements (Kennard, 1947; Gellhorn, 1953, 1967a; Hamm et al., 2003). Electrical stimulation of the hypothalamus (the principal controller of the ANS) is known to produce coordinated defensive or appetitive responses accompanied by signs of emotional arousal (Hess, 1925; Gellhorn, 1970) via projections to midbrain nuclei (PAG, locus coeruleus) and the pontine and medullary reticulum (Gellhorn, 1967a). Any activation of the muscular system also involves circulatory and cardio-respiratory adjustments mediated by the ANS; significantly for the PS concept, even imagined movement activates an autonomic response appropriate to the anticipated exertion (Collet and Guillot, 2010). The ANS is strongly linked to the subcortical affective centers, and there is evidence that each emotion has a specific autonomic signature (Ekman et al., 1983; Levenson, 1992; Hamm et al., 2003).

Early concepts of the ANS regarded it as a simple reciprocal bivalent system composed of a sympathetic and a parasympathetic division, which stimulated or calmed visceral functioning (Hess, 1925). Hess (1925), an early researcher into the functioning of the hypothalamus, recognized that the ANS did not function in isolation but was part of an integrated response including the motor system, the arousal/attentional system, and neuro-humoral response. He used the terms ergotropic (energy-seeking) and trophotropic (nourishment-seeking) to refer to the integrated functioning associated with the sympathetic and parasympathetic systems respectively. These terms have fallen into relative disuse, but they facilitate description of our concept of unitary subcortical action, and we will use these terms in this paper.

The bivalent view of the ANS has been replaced by a view of the ANS as being capable of complex and nuanced responses to varied circumstances (Saper, 2002). Gellhorn (1970), Levine (1977), and Berntson (Berntson et al., 1994) demonstrated that sympathetic and parasympathetic branches were not limited to reciprocal action; the could vary independently, or co-vary in the same direction. Saper has shown that the hypothalamus is capable of orchestrating complex autonomic responses to varying physiological demands such as thermoregulation, hunger and thirst, and a range of emotional states (Saper, 2002).

Porges (2007) has suggested a three-fold division of the ANS: (a) the evolutionarily primitive, dorsal vagal system that promotes immobility and shut-down, (b) the sympathetic system that mobilizes the fight-or-flight response, and (c) the ventral vagal system that facilitates social engagement. On this basis Porges identifies five global states, involving varying combinations of these: relaxed social engagement, vigorous social play, aggressive/defensive mobilization, relaxed resting immobility, and fear-based freeze or collapse (Porges, 2001). Each of these can be seen as a form of PS, involving coordinated autonomic, motor, affective, and cognitive states.

Interoception is the name given to the perception of information coming to the brain from the viscera (Vaitl, 1996); this also includes afferents from skin (Björnsdotter et al., 2010) and fascia (Schleip, 2012). Craig and Critchley have delineated the pathways whereby some of this information can reach consciousness and bring information about affective and autonomic state (Craig, 2002; Critchley et al., 2004) Damasio (Damasio et al., 1996) suggests that interoception may carry important information about the environment. It has been shown that even unconscious visceral afferents can strongly influence affective experience and behavior (Ádám, 1998). Training can enhance conscious awareness of interoception (Holzel et al., 2011b; Farb et al., 2013) and thereby increase emotional self-regulation (Vago and Silbersweig, 2012).

#### Emotional/motivational response

A widely held view is that emotion is strongly related to a disposition toward action (for instance (Bull, 1945, 1951; Lang et al., 1990, 1993; Frijda, 2004) Bull's attitude theory of emotion states that emotional affect emerges out of the preparation for action rather than the action itself (Bull, 1951, 1955). Her investigation of hypnotically induced emotion demonstrates that the preparatory movement responses to emotion constitute an integral aspect of the experience of emotion. If a certain preparatory movement state was (hypnotically) suggested to the subject (for instance, leaning back in the chair), they were unable to experience a (hypnotically suggested) emotion that contradicted this (for instance, anger) (Bull and Gidro-Frank, 1950). De Rivera (1977) extended Bull's work, offering a conceptual schema using five dimensions of movement to characterize a broader range of human emotional experience. His schema has not been investigated, beyond one study of consistency and reliability, but his ideas suggest the possibility of more detailed correlations between emotion and movement preparation. One possible line of investigation would be to produce abstract animations embodying the dynamics suggested by De Rivera (“move myself away from the other person,” “move the other person away from me,” etc.) and discover whether subjects attribute emotional motivation to the images.

Panksepp identifies seven core emotional systems (Panksepp, 2005), each associated with specific subcortical nuclei and neurotransmitter profile. He regards the motoric and affective aspects of these as equally important Each emotional system involves motivational feeling as well as characteristic movement and movement preparation patterns, supporting our hypotheses. He argues for these systems as the source of both human and animal basic emotions; his arguments are as follows.

- Direct brain stimulation in animals and humans produces strong unconditioned emotional reactions, whereas cortical stimulation has much less effect.
- The locations which produce such emotional responses are in homologous areas in all animals tested.
- All primary emotional responses remain intact after radical de-cortication early in life.
- In humans, severe damage to the insula, the visceral/affective cortex, does not eliminate emotional experience
- One can demonstrate that animals like or dislike stimulation of these areas, which can be used as reward and punishment
- When homologous brain regions are stimulated in humans, subjective experiences are reported which correspond with the animal behavioral responses.

For a detailed exposition see (Panksepp, 1998, 2005, 2011).

#### Attentional response (awareness)

Attending is an act as well as a receptive experience; and there is a spectrum from purely reflexive to fully volitional attending. Fan (Fan and Posner, 2004) proposes a distinction between alerting, orienting, and executive direction of attention. The initial alerting reflex is automatic and unconscious. It involves hippocampus, superior and inferior colliculus and the locus coeruleus, PAG, ventromedial medullary reticular formation, and medullary nuclei controlling head movement (Radulovaćki and Adey, 1965; Öhman et al., 2000). At this level a rapid automatic evaluation is already being made (Porges, 2004) and we suggest that the PS begins to be organized. Next a more complex and nuanced orientation process begins, directing the appropriate senses in the appropriate directions, orienting to available resources, and completing the PS process. More rostral areas become involved later: the parietal lobe and the frontal eye fields (for visual stimuli), and finally, with volitional direction of attention, anterior cingulate and prefrontal cortex (Fan and Posner, 2004). The exact point at which this process becomes conscious is not clear. We suggest a continuum of awareness from completely unconscious and automatic to fully conscious and voluntary, and that the training of voluntary attention may lower the threshold so that previously automatic responses may come under voluntary influence. Such alterations have been demonstrated, see for instance (Brown et al., 1984).

Affect influences attention at a fundamental level: for instance, negative emotions narrow and focus the field of attention in specific ways, whereas positive emotions open the attentional field beyond that of neutral attention (Fredrickson and Branigan, 2005). We hypothesize that this influence goes in both directions: voluntarily broadening the field of attention may induce a more positive affective state. BTES make such claims, which could be tested as described below.

The PS has been explored extensively in the context of simple perceptual attentional tasks, involving the study of anticipatory saccadic eye movements (Ruge et al., 2013). These studies are of limited relevance here as they deal with very short time frames and affectively neutral situations. Brunia points out the strong relation between anticipatory attention and motor preparation, and suggests that the reticular nucleus of the thalamus has a central role in both (Brunia, 1999). It has been shown that anticipated action mobilizes the ANS in accordance with how much effort is anticipated; and that uncertainty increases the amount of activation (Brunia, 1993). This supports our hypothesis of the link between attention and motor and autonomic state, suggesting the possibility (as claimed by the BTES) of altering muscle tension, posture and autonomic arousal by voluntary modulation of attention. Meditation has been studied primarily as an attentional strategy, and its positive effects on autonomic, affective and cognitive processes are well documented (Raffone and Srinivasan, 2010; Chen et al., 2012; Desbordes, 2012; Fox, 2012; Friese et al., 2012; Kox et al., 2012; Sedlmeier et al., 2012; Vollestad et al., 2012; Tang et al., 2014).

#### Expectation: cognitive response, appraisal, implicit memory

The term “cognitive” can be ambiguous (Canamero, 1998). While it refers broadly to knowledge and awareness it may conflate different kinds of knowledge. It may confuse explicit fully conscious verbal and conceptual knowledge with implicit, emotional, non-verbal knowledge. This distinction has recently been explored by Stott (2007) as “rational-emotive dissociation.” In memory research a clear distinction is made between explicit and implicit (Schacter et al., 1993). Explicit (autobiographical and episodic) memory can be brought into full consciousness, considered and re-evaluated. Implicit (including procedural) memory usually cannot, and is stored in different parts of the brain (Reber, 2013). Implicit memories influence behavior without one being conscious of this influence (Roediger, 1990). Procedural memory stores the action patterns associated with different situations and activities. The appraisal of events is largely based on memory (Glenberg, 1997). If that memory is explicit (subject to conscious thought and decision), we could term it explicit appraisal; if the memory is implicit it is implicit appraisal (Castelfranchi, 2000; Stott, 2007), or what Polanyi termed “tacit knowledge” (Polanyi, 1962).

Implicit and procedural memories are quickly activated and are accessed at a subcortical level (Roediger, 1990). They activate a set of expectations about the situation. Glenberg (1997) suggests that the main function of memory is to guide appropriate action (and therefore preparation for action) in the present moment. The simulation of bodily experienced states and actions are significantly involved in memory (Ross et al., 2007), understanding (Barsalou et al., 2003; Pulvermüller, 2005), interpersonal communication (Hostetter and Alibali, 2004), social interaction (Sebanz et al., 2006), and spatial perception (Tversky, 2003). This supports the idea that the expectancy aspects of the PS are intrinsically linked with affect and bodily preparedness.

Fully conscious appraisal involving explicit memory is slower, and comes after the rapid initial appraisal. Although cortical processing can and does modify the activity of the subcortical centers (“top-down”), the “bottom-up” connections are more extensive and arguably at least as powerful (Critchley, 2013). People frequently find themselves reacting and deciding “emotionally,” often based on early implicit learning (Hovdestad and Kristiansen, 1996; Luethi et al., 2008; Orange, 2011). Appeals to “reason” concerning emotional reactions are often ineffective (Langer, 1975; Langer et al., 1978; Goleman, 2006; Goel, 2014). The theory of cognitive dissonance states that, once a certain set of expectations is in place, people tend to ignore disconfirming perceptions and notice confirming ones (Festinger, 1962).

Appraisal at a subcortical level has a direct and immediate effect on affective, visceral and motor function, and is not easily accessible to change (Critchley, 2013). Cortical (explicit) appraisal is easy to access consciously, fairly easy to change, but may not have much effect on emotion and autonomic state (van der Kolk, 2002).

We suggest that subcortical, implicit appraisal is a core aspect of the PS, to be distinguished from fully conscious rational appraisal. In this paper we will use the term “expectancy” or “expectation.” We define this as a rapid, automatic, marginally conscious process of appraisal, accessing implicit and procedural memory and generating a set of expectations concerning the present situation. Porges' concept of “neuroception” (Porges, 2004) is an example of such a process. He postulates a largely unconscious, sub-cortically processed perception of the safety, or lack thereof, of the environment.

### Categories of PS

The concept of PS leads to the question: what different kinds of PS are there? The early view of the ANS as bivalent (ergotropic and trophotropic) suggests two PSs: readiness for active mobilization, and preparedness for quiet recuperation.

Levine (1977) and Berntson (Berntson et al., 1994) have demonstrated that the parasympathetic and sympathetic branches are not limited to reciprocal activation, suggesting a range of possible states.

Porges' polyvagal theory suggests five states (based on combinations of dorsal vagal, sympathetic, and ventral vagal activity): immobility without fear (dorsal vagal) immobility with fear (dorsal vagal and sympathetic); fight-or-flight mobilization (sympathetic), play (sympathetic and ventral vagal), and social engagement (ventral vagal).

Panksepp defines seven core subcortical emotional circuits, each of which is neurologically and chemically distinct and has its own characteristic postural, affective, autonomic, attentional, and cognitive patterns: SEEKING, FEAR, ANGER, LUST, NURTURANCE, PLAY, GRIEF (Panksepp, 2011) (The use of capitals is Panksepp's way of distinguishing his proposed classification from the ordinary use of these words). This schema partly overlaps Porges', and could form the basis for a partial taxonomy of PSs. The instinctive appetitive drives such as hunger, thirst, and thermoregulation, could also define specific PSs. Our theory suggests that any of these PSs (a preparatory state oriented to escape, attack, sexual activity, exploration, nurturance, etc.) could become maladaptive through persistence or disorganization, and form the basis for various kinds of distress, not all of which would currently be termed “stress.”

### Stress and the PS

How is the PS relevant to stress, and how does this differ from the view that stress is excess sympathetic arousal? Cannon's (1939) and Selye's (1954) early theories of stress hypothesized a unitary “stress response” involving a series of automatic neurophysiological reactions: activation of the sympathetic and inhibition of the parasympathetic nervous systems, triggering of the adrenal medulla and cortex, the release of catecholamines and cortisol. More recent research reveals the complex modulation of this response in the face of different forms of physiological challenges (Saper, 2002), the complex dynamics of the interaction between sympathetic and parasympathetic branches of the ANS (Gellhorn, 1967b; Levine, 1977; Berntson et al., 1994), the interactions between the ANS, the immune system (Mignini et al., 2003), and other subcortical structures modulating arousal, attention, affect, motivation, and movement (Tucker et al., 2000; Öhman et al., 2000; Strominger et al., 2012; Leventhal, 2014; Norman et al., 2014), and the neurochemical details of allostatic load and overload (Seeman et al., 2001; Lupien et al., 2006), as well as the involvement of cortical structures in these subcortical processes (Berntson and Cacioppo, 2007). Despite these developments the single word “stress” continues to be used. To our knowledge, our hypothesis that stress can be understood in terms of preparation for action has not previously been proposed.

Stress is not a single phenomenon, and we believe that the use of the word in a scientific context is no longer justified. Instead we suggest that the broad range of phenomena subsumed under the term can be discussed in terms of whether the current PS is adaptive or maladaptive to the current situation. Some ambiguity may be involved, in that there could be more than one kind of response that might be adaptive in certain situations.

This framework allows the discussion of all the phenomena referred to as stress, the clarification of the five questions about stress raised above, and the discussion of a number of dysfunctional states not normally referred to as stress. In addition it allows the consideration of healthy and optimal responses. The discussion below focuses mainly on ergotropic and trophotropic PSs; we hypothesize that similar dynamics are involved in all PSs, but there is as yet little data to support this.

#### Adaptive PS

The simplest form of stress is sympathetic (ergotropic) arousal in a challenging situation: fight or flight. This is necessary, appropriate, and not problematic. In response to moderate acute challenge, there is a rapid increase in sympathetic activation accompanied by a decrease in parasympathetic activity. This ergotropic state facilitates vigorous response to the challenge. This is followed by a parasympathetic “rebound,” and a return to baseline of the sympathetic activation (Gellhorn, 1956). This is usually referred to as acute stress (Everly and Lating, 2013). In PS terms, the adaptive PS leads to effective action, the situation is successfully resolved, and the PS of arousal subsides and is replaced by one of recuperation (trophotropic). This process occurs both in sudden threatening situations, and also in voluntary activities such as sports and risky recreation. This short-term activation of adaptive ergotropic PS has been called “good” stress (Everly and Lating, 2013). It usually leads to increased ability to handle stress: “stress inoculation” (Meichenbaum et al., 2009) or resilience (Karatsoreos, 2013; Wu et al., 2013). We hypothesize this involves an increased ability to adopt *and relinquish* PSs. This could be tested by observing whether exposure to successfully resolved moderate stress increases performance on a “stop signal” task (Logan, 1994) (which measures the ability to let go of expectations).

However, if the threatening situation is prolonged, negative neurophysiological consequences [allostatic overload (Lupien et al., 2006)] may occur despite the adaptive nature of the PS. This is one form of chronic stress, which can only be prevented by ending exposure to the challenging event. If the situation is very extreme, it may exceed the realistic capacities of the organism to cope. In this case, the PS may nevertheless remain coherent and organized, in which case there may be few lasting consequences once the event is over. Or the PS may become disorganized (competing PSs may be simultaneously aroused—see below for discussion), which increases the chance of post-traumatic stress (PTS) (Bovin et al., 2008) (See Table 1).

**Table 1:** **Outcomes of adaptive PS in four situations**.

**PS**	**Action**	**Result**	**State of PS**	**End result**
Adaptive PS in time-limited situation	Appropriate action taken	Resolution of situation	Release of PS	Formation of new PS. Increased Resilience
Adaptive PS in chronic situation	Appropriate actions taken	Situation not resolved quickly	Prolonged PS	Prolonged PS—Increased AL; possible eventual overwhelm
Adaptive PS in overwhelming situation	Appropriate actions taken	Situation overwhelming	PS stays organized	Once the situation ends, no PTS
Adaptive PS in overwhelming situation	Appropriate actions taken	Situation overwhelming	PS disorganizes: trauma	Once the situation ends, likely PTS

When the PS is well matched to the challenging situation (adaptive), there can be a variety of outcomes depending on the situation. If the situation is successfully resolved within a short time, there will be increased long-term resilience. If the situation fails to resolve quickly, there may be increased allostatic load. If the situation overwhelms the capacity to cope but the PS stays organized, there may be few long-term consequences. However if the PS becomes disorganized (see text for explanation) there may be lasting post-traumatic stress. (AL, allostatic load; PTS, post-traumatic stress.)

#### Maladaptive PS

We define four kinds of maladaptive PS; see Table 2 below. First, the PS could be well-organized but not well matched to handling this particular situation. Second, the PS itself could be disorganized. In either case, the maladaptive PS may be “persistent” (a PS which arose in response to an earlier situation but has continued beyond its utility) or “situational” (a PS in response to the present situation which is not well adapted to handling that situation. By definition, a PS is organized to enable effective action. Once the action is successfully completed, the PS subsides. We suggest that one cause for a PS not subsiding (persisting) is the failure to complete the action pattern. Mandler's experiments on the interruption of organized behavior as a principal cause of anxiety and disorganized behavior are supportive of our suggestion (Mandler, 1964; Mandler and Watson, 1966).

**Table 2:** **Two dimensions of maladaptive PS**.

**Maladaptive PS**		
Situational	Organized	Disorganized
Persistent	Organized	Disorganized

*Maladaptive PSs can be categorized in two dimensions. First, whether they have arisen in (poorly matched) response to the present situation, or whether they are chronic, persisting unresolved from a previous situation. Second, whether (despite being maladaptive) they are organized (retaining coherent goal organization), or whether they are disorganized (competing PSs arising simultaneously)*.

##### Persistent PS

If ergotropic arousal [in Panksepp's terms, the FEAR and RAGE circuits (Panksepp, 2011)] persists long after the situation has passed, this is problematic. The PS does not end, and the organism continues to prepare for challenge. Ergotropic arousal is designed to handle time-limited challenges and has deleterious neural and neurochemical effects (“allopathic load”) if sustained. Since the challenging situation is no longer present, action cannot be taken and the PS does not resolve (Mandler and Watson, 1966). This usually leads to decreased resilience as allopathic load increases (Seeman et al., 2001).

The question “what are the neurochemical details of allopathic load” is different from “what causes the PS (and thus the accumulation of allopathic load) to persist?” The PS perspective distinguishes the *effects* of “stress” from the actual nature of “*stress*” *itself*: the maladaptive PS. In our view, it is the ending of the maladaptive PS that ends the core stress response, not the correction of neuro-humoral imbalance or the removal of the challenging situation. (Of course, correcting neuro-humoral imbalance due to disease or genetics, and remedying the external situation, are crucial where possible.)

Gellhorn has shown in rats that a shock above a certain threshold of duration and intensity prevents the normal parasympathetic rebound, and the sympathetic system may remain activated indefinitely (persistent PS). He refers to this as “tuning” (Gellhorn, 1968). A similar phenomenon, heightened reactivity of the amygdala, is termed “kindling” (Cottrell and Nyakas, 2013). We suggest that both are examples of persistent PS.

**Figure 2:**
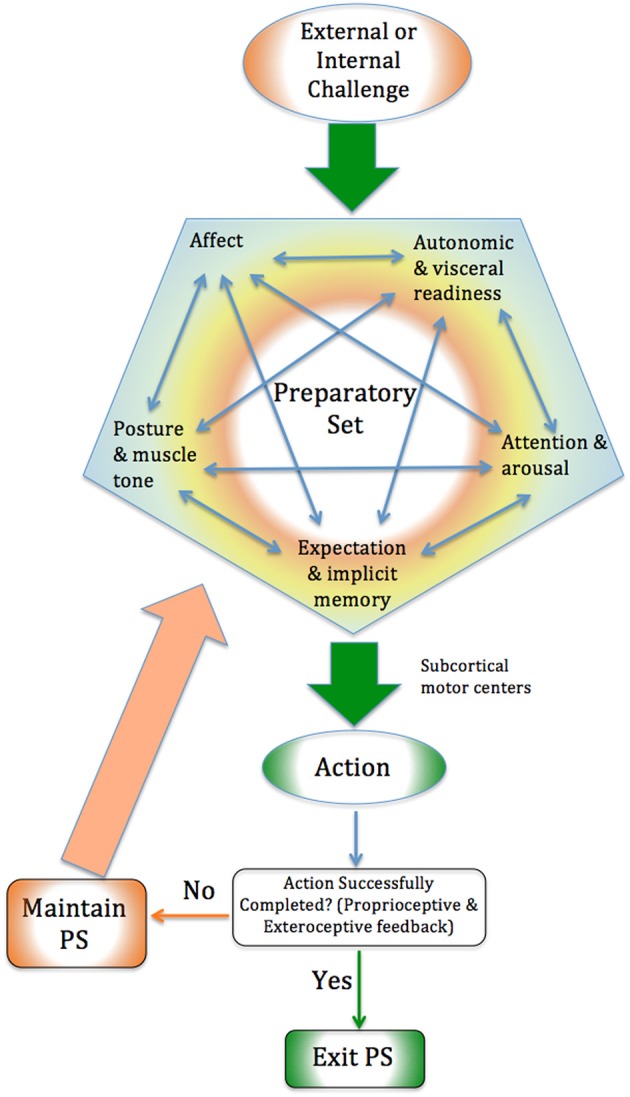
**Biological Completion In response to situational challenge, the PS prepares a response**. If the preparation culminates in successful action, the PS can subside. If not, the PS may persist. Proprioceptive and exteroceptive sensory information informs the organism about the success or completion of the action.

If this hypothesis is correct, it would suggest that if the rat is given an opportunity to complete the PS in vigorous action, the PS would be released and the behavioral problems disappear. This has been shown to be the case. A rat alone in a cage may develop behavioral problems in response to shock; but if the rat is in a cage with another rat with which it can fight, it is much less likely to develop problems (Weinberg et al., 1980). In humans, subjects startled by a pistol shot but instructed not to move remained in a state of elevated sympathetic activation afterwards, but if encouraged to move vigorously their system returned to normal (Freeman and Pathman, 1942). If rats are subjected to shock but restrained from escaping, they develop behavioral problems (Mandler and Watson, 1966). If the rats are later placed in the original situation and given an opportunity to escape through vigorous action, the conditioned fear rapidly disappears (LeDoux and Gorman, 2001). We suggest this is because the PS is now given the opportunity to resolve and the action patterns can complete. These results suggest that if adequate ergotropic activity does not occur the ANS may not return to balance and the PS generated in response to the threat may persist. This supports our hypothesis that completing the action pattern of a PS [“biological completion” (Levine, 2010; Payne et al., 2015)] enables it to subside (See Figure 2 for a summary).

##### Disorganized PS

If the threat is extreme, inescapable, or very prolonged, there may be an apparent “spillover” of activation from the ergotropic to the trophotropic systems, possibly due to spreading neural arousal from anterior to posterior hypothalamic nuclei (Gellhorn, 1967a). Several researchers have documented this phenomenon (Gellhorn, 1959, 1967a; Paton et al., 2006). It involves simultaneous high activation of sympathetic and parasympathetic systems and the loss of effective coping [“co-activation”: see (Berntson et al., 1994)]. Such a reaction can be induced in animals by physically restraining them from escaping shock (Mandler and Watson, 1966; Shors et al., 1989; Amorapanth et al., 2000). This is the canonical way of inducing PTSD-like symptoms in animals. This response has been called tonic immobility (TI) (Hoagland, 1928; Bovin et al., 2014; Marx et al., 2008). TI and similar symptoms have been associated with increased likelihood of PTSD in humans, and develop under similar conditions of restraint (Suarez and Gallup, 1979; Volchan et al., 2011; TeBockhorst et al., 2015). TI prevents effective completion of the defensive response. Porges' polyvagal theory characterizes this fear-based immobility as a simultaneous activation of the sympathetic and the evolutionarily more primitive dorsal vagal system (Porges, 2001). We suggest this can be conceptualized in our framework as the simultaneous arising of mutually incompatible PSs (flight and freeze), which leads to a disorganized and ineffective response. We suggest that the ensuing “stress” and behavioral problems can be explained as the maladaptive persistence of the disorganized and interrupted PSs. This may provide an alternate way of viewing “dissociative” behavioral phenomena. The PS perspective suggests that resolution of these symptoms can come about by enabling the PSs to be released through completion of the action pattern. However, this resolution may not happen as readily in animals with TI as with sympathetically “tuned” animals: when animals with TI are placed again in the cage they may not spontaneously try to escape. The researcher may have to physically move them to safety before the instinctive defensive action will take place (Gellhorn, 1967c), indicating a clear distinction between what we are calling “organized” and “disorganized” PS. Our theory suggests that in such cases two contradictory PSs must be released before resolution can take place: the escape and the immobility. This suggests greater clinical difficulty in dealing with the latter, which is widely recognized to be the case. See Table 3 for further discussion, see below under Biological Completion, also (Levine and Buczynski, 2013; Payne et al., 2015).

**Table 3:** **Four kinds of maladaptive response and treatments suggested by PS view**.

**PS**	**Organized/disorganized**	**Reason for maladaptation: Situational or persistent**	**PS perspective on effective treatment**
Maladaptive PS	Organized PS	Faulty subcortical appraisal of present situation (situational)	Reappraisal through awareness of PS—voluntary re-organization
	Organized PS	Held over from a previous unresolved situation (persistent)	Resolution or release of previous situation
	Disorganized PS	Overwhelmed by present situation (situational)	Trauma first aid and treatment needed
	Disorganized PS	Overwhelmed by previous unresolved situation (persistent)	PTSD or DESNOS: Resolution of past trauma needed

The PS perspective suggests that the four kinds of maladaptive PS (organized/situational, organized/persistent, disorganized/situational, disorganized/persistent) each call for a different therapeutic response. If the PS is well organized and arises in response to the current situation, but is poorly matched, a process of conscious re-organization may be effective. A well-organized but persistent maladaptive PS could be termed a “bad habit”; a resolution through increased awareness of the origins of the PS may help. If the PS is disorganized, and the direct result of the present situation, immediate trauma “first aid” may be very effective. If on the other hand the disorganized PS is the result of past unresolved experiences, PTSD or “disorders of extreme stress not otherwise specified” (DESNOS) may follow, which require specific trauma-oriented therapy. DESNOS is a term from DSM V which covers a variety of trauma sequelae which fail to fall into the category of PTSD. (DESNOS: Disorders of Extreme Stress Not Otherwise Specified)

#### Varieties of “stress”

As discussed above, one advantage of the PS framework is that it enables identification of a wide range of specific dysfunctional conditions. These conditions may be referred to collectively as “stress,” even though they are quite distinct; or they may be excluded from this category despite the fact that it would not be true to claim they are “not stressful.” We suggest that *any* maladaptive PS, is problematic for the organism, not only ergotropic PS involving anger and fear. We suggest that different maladaptive PSs will have discrete harmful consequences on all levels from physiological to psychological. Animal experiments aimed at frustrating specific drives could test this.

We further suggest that our model of disorganized PSs could apply more broadly than in the above example of conflicting immobility and escape impulses. We suggest it might be possible for extreme activation of any PS to spread into another, competing PS, and cause disorganization and ensuing behavioral problems. In animals, this could be tested through direct brain stimulation.

Following Panksepp's model of seven primary emotional states, each with its own neural and neuro-chemical substrate (Panksepp, 2011), we suggest that a persistent FEAR PS will have different neurochemical, neural, and psychological consequences from a persistent RAGE PS. Likewise, the intrusion of a NURTURANCE PS into a situation that requires RAGE, a LUST PS when NURTURANCE is required, a GRIEF PS when SEEKING is required, each will have different negative consequences, each with a distinct profile. It is hardly useful to call them all “stress,” nor to debate which constitute “stress” and which not. We believe the PS framework offers a more precise and nuanced way of characterizing a wide variety of problems, and could guide targeted therapeutic approaches. Bos and Panksepp have already used a similar framework to suggest novel pharmaceutical approaches to different forms of stress (Bos et al., 2012). The identification of the specific PS involved in particular forms of emotional or autonomic dysfunction could guide effective intervention via BTES or cognitive-behavioral therapies. A nuanced understanding of maladaptive PS, could increase the effectiveness of a wide range of medical diagnoses and interventions.

#### Five questions about stress

Below we restate the five questions about stress posed at the beginning of the paper, and indicate briefly how the PS perspective can clarify them. Many of these issues have already been addressed above and are summarized below.

##### “Good” stress/“bad” stress

The mobilization of organismic resources in response to challenge is necessary and beneficial, and yet under some circumstances mobilization can lead to significant problems. A distinction has been made between “good” stress and “bad” stress (Selye, 1985), but the exact nature of the difference between them is not clear. The PS perspective distinguishes adaptive and maladaptive PSs; the former generally lead to greater resilience (“good” stress), the latter to various harmful consequences (“bad” stress).

##### Acute, chronic, and traumatic stress

Stress has been classified as acute, chronic and traumatic. This classification has been made on the basis of differing neurophysiological reactions (Everly and Lating, 2013); but it remains unclear what determines these differing responses. The PS perspective suggests that acute stress involves an appropriate, time-limited PS appropriate to threat or challenge (mobilization of active resources); chronic stress involves either a persistent maladaptive PS or adaptive response to prolonged challenge; and traumatic stress involves a disorganized PS characterized by regression to a phylogenetically more primitive (Porges, 1995) form of PS (Halvorsen, 2014).

##### What is resilience?

It is widely recognized that different people exposed to almost identical events can respond in very different ways. It has been shown that the presence or lack of resilience may be due to genetic factors, adverse childhood experience, and personality make-up (Karatsoreos, 2013). However, there is little clarity about exactly what this resilience *is*.

The PS perspective suggests that resilience is in part the ability to let go of inappropriate PSs and to adopt functionally appropriate ones. Adverse childhood experience may cause persistent maladaptive PSs, and some measures of personality associated with poor resilience may be measures of maladaptive persistent PSs. We suggests that resilience may be improved by ending persistent maladaptive PSs as well as by learning new adaptive ones. We also suggest that cognitive techniques will be effective only to the degree they influence subcortical organization (van der Kolk, 2002). This suggests the value of methods (such as those used by BTES) which more directly affect subcortical areas.

##### Appraisal and expectation

A distinction has been made between physical and physiological stressors (such as cold or toxin) and psycho-social stressors (such as a challenging social situation or a negative thought) (Everly and Lating, 2013). In the latter, the person's appraisal (and consequent expectations) of the situation is regarded as largely determining the response (Olff et al., 2005). However, the organism's response even to physical stressors can be altered voluntarily. For instance Hof and volunteers trained by him have been studied for their anomalous physiological responses to extremes of cold exposure and inflammatory challenge (Gard et al., 2014; Riley and Park, 2014). A significant part of this training involves the re-appraisal of the nature of cold as well as BTES techniques of breath control, posture and imagery (Kox et al., 2012, 2014).

On the other hand the concept of the “appraisal” of a psycho-social stressor is not clear. The mere thought that a situation will not be stressful is unlikely to change a person's response; and yet the previous examples, as well as the proven effects of cognitive restructuring therapy, show that appraisal can have a significant impact. Why should appraisal sometimes appear to determine outcome and at other times not?

The PS perspective suggests that re-appraisal occurring only on a verbal/conceptual level is less likely to alter the PS, and thus the stress response; but altered subcortical expectations may be more effective. This suggests that attention to interoceptive and proprioceptive experience may be more effective in handling stress than attention to ideas and thoughts alone, because bodily experience enables more direct access to the PS.

##### What is the stress response itself?

Recent work on “allostatic load” (Cicchetti, 2011), the accumulated “wear and tear” from continual adjustment to changing life conditions, has provided detailed knowledge about the neurochemical and neuroplastic *effects* of stress. In our view, this still leaves unaddressed the core of what the stress response itself *is* and how it can best be changed.

The PS perspective suggests that the essence of the stress response is the perpetuation of maladaptive PSs, and that effective treatment of stress should involve a focus on changing subcortical preparatory organization. The BTES offer methods with this focus.

## BTES

### What are BTES and how do they relate to PS?

BTES present a specific challenge to the scientific investigation of human suffering and its remediation. Traditional science has been based on the Cartesian dualism of mind and body (Damasio, 2005), whereas the BTES are firmly based in a non-Cartesian view. Despite the current interest in “mind-body medicine,” this paradigm persists, as does the continuing gulf between the BTES and mainstream medical and psychiatric theory (Leder, 1992). In presenting the PS as an explanatory framework we hope to demonstrate that current neuroscience supports the view of the BTES that bodily aspects (movement, posture, proprioception, interoception) are, at a basic level, inseparable from affective and cognitive aspects of the organism.

#### Defining BTES

We define BTES as a diverse group of educational and therapeutic practices, of traditional Asian and modern Western origin, which share a common set of assumptions and practices. They encourage attention to posture, breathing, movement, and proprioceptive and interoceptive sensation, as well as tactile and spatial awareness; and they use this bodily attention as well as voluntary movement and imagery as the principal means to accomplish therapeutic change (Read and Stoll, 2009). They do not deny “disease,” but do not use a disease framework in evaluation or treatment (This distinction can be a central issue in studies of efficacy related to medically defined disease states.). Importantly, BTES do not make a fundamental distinction between body and mind, tending to regard them as poles on a continuum rather than fundamentally different realms (Rosenthal, 1991). Our hypothesis is that the PS is an integrated subcortical state that is significant in physical and emotional distress, and that interventions on any one of its components will tend to alter all the components. Further, that BTES interventions are based on this view; see Table 4. At the end of this article we summarize the suggestions we have made for testing these hypotheses.

**Table 4:** **Use of PS in BTES**.

**BTES**	**References**	**Components of PS**				
		**Posture/tone**	**Autonomic**	**Affect**	**Attention**	**Expectation**
Qigong	Cohen, 1999	P	P	P	P	P
Yoga	Satchidananda, 1978; Sivananda, 2005; Singleton and Byrne, 2008; Jois, 2010	P	P	C	P	P
Meditation	Johnson, 2000; Frantzis, 2001; Kabat-Zinn, 2005; Dorjee, 2013	P	C	C	P	P
Alexander	Jones, 1976; Alexander and Maisel, 1989; Gelb, 1995	P	C	C	P	P
Feldenkrais	Feldenkrais, 2002, 2005; Rywerant and Feldenkrais, 2003	P	C	C	P	C
Rolfing	Rolf, 1989; Sise, 2005; Karrasch, 2009	P	C	C	P	C
Reichian	Boadella, 1974	P	P	P	C	P
Formative	Keleman, 1971, 1979, 2013	P	P	P	P	P
SE	Levine, 1997, 2010; Payne et al., 2015	P	P	P	P	P

*The listed BTES use interventions that involve several of the components of the PS. Stated claims of influencing other components of the PS are also listed. Although the BTES differ in emphasis and in the details of the intervention, they share a similar framework. The references in this table are to substantiate that the BTES claim to use these forms of intervention and to have these effects; they refer mainly to the writings of the founders or prominent practitioners, not to peer-reviewed publications. Key: P, Principal form of intervention C, Effect claimed*.

##### BTES are normative

BTES aim to increase overall human health, physical, emotional and mental, toward optimal functioning, and not only to remedy ill health. They all include the concept of an optimal state which maximizes adaptive ability across a wide range of situations. This is generally defined (Johnson, 1995, 1996) as a state that is flexible, balanced, open, and capable of adjusting quickly to the varying demands of the environment. Such a state allows the rapid adoption of specific adaptive PSs as well as the ability to quickly relinquish them. It is variously described as involving proprioceptive experiences of balance, stability, and lightness (Jones, 1976); an open flexible attention (Brown and Ryan, 2003); interoceptive experiences of calm, warmth, and flow (Csikszentmihalyi, 2008); flexible expectations (Farb, 2012); and affective feelings of openness, confidence and curiosity (Ekman et al., 2005). BTES claim that optimal PS is quite rare, that most people are stuck in maladaptive PSs.

#### Changing PS

We have suggested that stress can be resolved by releasing the maladaptive PS, and that this is the primary aim of BTES. A broad examination of the BTES suggests that there are two principal ways PSs can be changed. The first is by becoming conscious of the process of maintaining the PS, voluntarily letting go of it and replacing it by a more adaptive PS. The second is through “biological completion,” in which the originally obstructed impulse is enabled, in a safe context, to complete itself.

##### Awareness of PS

Both of these processes, voluntarily letting go of a maladaptive PS and biological completion, require awareness of the PS. Since PS are primarily sub-cortical, it may not be easy for a person to be fully, reflectively aware of their PSs, especially in the case of persistent maladaptive PS. BTES claim that this lack of awareness is the major obstacle to being able to let go of the PS, and that by cultivating the capacity to attend to subtle aspects of proprioception and interoception (as well as attentional orientation and affective state) it is possible to become aware of the PS and of one's active (if involuntary) process of adopting and maintaining it.

Damasio's theory of somatic markers (Damasio et al., 1996; Damasio and Carvalho, 2013) emphasizes the important role of interoceptive awareness in becoming aware of one's inner state. Interoception enables one to become aware of the (autonomic) state of the body, and is strongly related to emotional response and affective and visceral aspects of the PS. Craig and Critchley (Critchley et al., 2004; Hölzl et al., 2009; Garfinkel et al., 2013; Sel, 2014), as well as earlier researchers (Newman, 1974; Mogenson et al., 1980), have suggested the neural pathways for this mechanism. The anterior insula and anterior cingulate cortex are where this information reaches full awareness. Proprioceptive and kinesthetic information likewise bring to consciousness other essential aspects of the PS including posture, muscle tone, and movement preparation (Suetterlin and Sayer, 2013). The cortex receives this information in the sensorimotor, pre-motor and supplementary motor cortexes (Gellhorn, 1964; Prochazka, 2011) as well as in the body schema area in the inferior parietal and extra-striate body area (Arzy et al., 2006; Daprati et al., 2010; Ionta et al., 2011). Proprioceptive and kinesthetic feedback bring information from the basic and EMS (Holstege et al., 1996), and thus can inform the conscious mind about unintended automatic or emotionally driven motion, bringing information about current PS.

##### Voluntary change

The BTES claim that once awareness has been achieved it is possible to alter the PS through the use of the following voluntary procedures:

- Proprioceptive or interoceptive imagery;
- Affective imagery;
- Adopting a specific posture;
- Performing specific movements;
- Breathing in certain patterns;
- Paying attention in certain ways;
- Modulating expectation/appraisal.

As indicated above, there is evidence for the effectiveness of each of these in altering stress responses. We suggest that the anterior cingulate (ACC) and the premotor cortex (PMC), as well as portions of the orbito-frontal cortex (OFC), are the principal originations of these actions. It has already been shown that ACC has significant effects on amygdala (Posner et al., 2007) and other subcortical autonomic, affective and motor areas (Critchley et al., 2005), as well being involved with acts of intention (Shenhav et al., 2013). PMC has rich connections to subcortical motor centers, alters posture, muscle tone and autonomic state (Jeannerod, 1994; Desmurget and Sirigu, 2009). OFC likewise has extensive connections to subcortical areas and is central to several executive networks (Price, 2007)}. It has been shown that both motor (Collet and Guillot, 2010; Anema and Dijkerman, 2013) and affective (Lang, 1979) imagery can alter subcortical activity.

##### Biological completion

In our discussion of maladaptive PS above, we referred to “biological completion.” This involves the completion, through imagery or safe re-enactment, of the impulses of the persistent maladaptive PS. Several forms of BTES use this approach, especially the more psychotherapeutically oriented ones. The term itself comes from Somatic Experiencing (SE), but many other BTES use methods others use methods which may involve this mechanism. This includes body-oriented therapies generally. Movement practices such as Spontaneous Qigong (Cohen, 1999), Kriya and Tandava Yoga (Odier, 2003), and T'ai Chi, may involve similar processes. Clinical experience in SE shows that biological completion often involves autonomic discharge such as trembling, flushing, or crying (Levine, 2010; Payne et al., 2015) which appear to result in a normalization of autonomic activity. These processes have been very little studied, although Gračanin (2014) has demonstrated that crying can facilitate autonomic and affective balance, and trembling, which has been documented during TI in response to rape (Suarez and Gallup, 1979), may be the beginning of such a discharge (Payne et al., 2015). Facilitating the completion of the defensive reaction, in a safe therapeutic context, restores balanced functioning to the ANS and the network of core subcortical centers, resolves the stress response, and allows the patient to let go of the trauma-oriented PS in which they had been stuck since the precipitating event. This completion happens through the use of imagery and subtle movement to enact a successful resolution of the situation. Biological completion is discussed at greater length in Payne et al. (2015) and Levine (2010).

We believe there is supportive evidence for this process; as mentioned above, of restrained animals recovering from trauma through completion of the escape response, as well as the role of TI in PTSD in humans. However this theory is still speculative. Systematized manualized application of this procedure in randomized controlled trials, with measurement of affective and autonomic variables, would help to clarify the impact of biological completion as compared with cognitive-behavioral and exposure therapies. It should be determined whether generalized vigorous movements have the same effect as specific completion of the movements involved in the original traumatic situation. In rats this might be tested by shocking and restraining the rat in a situation requiring a given response, then later offering some of the rats an opportunity to complete that response (e.g., running to safety), while offering others a quite different activity (such as climbing to get food). The biological completion hypothesis would suggest that the former group would recover more completely than the latter.

#### Research on BTES

Seated meditation (Raffone and Srinivasan, 2010; Travis and Shear, 2010; Holzel et al., 2011a) and Yoga (Gard et al., 2014) have received the most research attention, with Qigong (Jahnke et al., 2010; Payne and Crane-Godreau, 2013) a close third. Somatics and body psychotherapy have has the least research reported, although clinical and anecdotal evidence for their efficacy is evidenced by informal published accounts, the authors' experience, and personal communications from practitioners. Given their promise to support and optimize health, we hope our proposed framework might facilitate research into their mechanisms. Research into Somatics particularly has been hampered by poor quality of experimental design, inadequate controls, small study size, poorly standardized interventions and most significantly for our paper, a lack of full understanding on the part of researchers of the concepts and techniques of BTES (Kerr, 2002). See for instance this study contrasting the philosophical approaches of the Feldenkrais Method and conventional exercise (Wright, 2000).

### Examples of BTES

A complete survey of all forms of BTES is well beyond the scope of this paper. We will confine our discussion to several of the better-known systems. BTES fall into three broad categories:

- Modern forms of traditional Asian psychophysical practices. In this category we will discuss Qigong and Yoga as well as seated meditation.
- Somatics. This term refers to a variety of educational and therapeutic methods of recent (20th century) Western origin; here we will discuss the Alexander Technique, the Feldenkrais Method, Continuum, and Rolfing.
- Western forms of “body psychotherapy.” We will discuss Reichian Therapy and its off-shoots Bioenergetics, Formative Psychology, and Levine's Somatic Experiencing trauma therapy.

#### Modern forms of traditional asian psychophysical practices

##### Qigong and Taijiquan

“Meditative movement” (MM) has been proposed as a term referring to a broad range of Asian contemplative practices (Larkey et al., 2009) including Yoga, Qigong (“Chi Kung”) and Taijiquan (“Tai Chi”). There are however significant differences between Yoga and Qigong, so we will discuss them separately.

Qigong teaches balanced standing and movement accompanied by spatial, proprioceptive and interoceptive awareness (Cohen, 1999). It brings conscious awareness to the (mechanical) postural processes of grounding, orientation, and correct relation of the longitudinal axis to gravity (Cohen, 2012), thus promoting optimal postural preparedness and a sense of self-efficacy; and through the monitoring of breathing and other interoceptive information, muscle tone and movement (proprioception), and thought activity, brings about a calm, centered state in which one is responding to the actual present condition rather than preparing for past or future events. Further, it trains one to let go of inappropriate anticipatory tension, to maintain resilient flexible posture with balanced muscle tone, and to maintain this balanced preparatory state while moving in simple or complex ways, moving with a partner, or even simulating attack and defense (Diepersloot, 1997). One of the great benefits of martial forms of Qigong (like Taijiquan) is that it teaches one to prepare for extreme challenge by remaining flexible, grounded and aware rather than tensing in fear or anger or collapsing in fear. Although Qigong practice may not specifically address past traumatic memories, we suggest it may facilitate processing such memories by retraining the dysfunctional PSs associated with them. Qigong practice characteristically involves using imagined movement to create very subtle changes in bodily posture and proprioceptive experience. This engages the premotor areas (Gerardin et al., 2000), central to our concept of the PS. The methods of Qigong are fully congruent with our hypotheses concerning the PS.

Research into Qigong has been hampered by poor experimental design, small studies, and difficulty translating the traditional theory into scientific terms; for a discussion, see (Kerr, 2002; Payne and Crane-Godreau, 2013). However most studies point to positive results in a wide range of physical and psychological conditions, in some cases even when compared to standard treatments (Ng and Tsang, 2009; Jahnke et al., 2010; Lee et al., 2011).

##### Yoga

Yoga has been shown to be of benefit in a wide variety of conditions, and its literature is quite extensive. For a recent review, including extensive speculation on mechanisms, see Gard (Gard et al., 2014). Hatha Yoga (the principle form of Yoga practiced in the West) uses specific postures, often held for periods of time, to stretch and strengthen the body and to focus the mind (Iyengar, 1966; Singleton and Byrne, 2008; Jois, 2010). In our theory, persistent PSs will involve chronic muscle tension. It is widely accepted in Yoga and the manual therapies that chronic muscle tension results in a shortening of the fascia (Chaitow, 2010). Yoga postures restore appropriate length and alignment to the musculoskeletal structure, which may provide a new proprioceptive experience which may in turn help establish more flexible PSs. Carney's research supports the effect of posture on affect and neurochemical secretion (Carney et al., 2010). Yoga also uses breath control to alter the autonomic state (Brown and Gerbarg, 2005a,b) as well as the control of the attention. Yoga explicitly aims at establishing a more optimal overall psychophysical state (Jois, 1999). This is consistent with our hypothesis that Yoga addresses the PS through intervention on its components.

##### Meditation

Meditation, in particular “mindfulness” meditation from the Buddhist tradition, has been studied extensively. However, the focus has been mostly on cognitive and attentional aspects of meditation (Raffone and Srinivasan, 2010; Travis and Shear, 2010; Holzel et al., 2011a). A distinction has been made between static seated meditation (such as the traditional Buddhist Shamata/Vipassana practices) and “movement meditations,” such as traditional Chinese Taijiquan (Larkey et al., 2009). While it is true that some forms of meditation emphasize primarily cognitive processes, the emphasis on posture in meditation is so widespread and so basic that this distinction must be questioned. In this paper we include seated meditation in the BTES category unless it places *no* significant emphasis on posture, breathing or physical sensation.

Although some authors have looked at the body awareness cultivated in some forms of meditation (Kerr et al., 2013), a crucial aspect has been almost completely ignored in the literature: posture. In almost all traditional systems of meditation, correct posture is stressed as an absolutely essential component (Johnson, 1996). In seated meditation, upright balanced posture is intrinsic to the meditative attitude (Johnson, 2000). We suggest that meditative awareness is a specific PS. In mindfulness, one prepares to meet each new experience without grasping, without shrinking away, but with flexible open presence, a balanced posture of mind, body and emotion. We believe meditation can be understood as a PS, a specific state of preparedness characterized by the absence of fixed maladaptive PSs.

#### Somatic approaches

##### Alexander technique

The Alexander Technique (AT), developed by F.M. Alexander in the early 20th century (Alexander and McGowan, 1997), is a method for changing habitual patterns of posture and movement, and claims far-reaching effects on physical and psychological health. It points to the prevalence of poor habit patterns, but goes beyond a purely physical focus to define these faulty habits as “poor use of the self”; the self is defined as “the entire psycho-physical organism” (Alexander and Maisel, 1989). These unconscious patterns of behavior involve attention, thought and emotion as well as the body The principal method involves helping the student to become aware of their subtle preparatory physical and mental tensions; learning to pause and to “inhibit” these tensions; and then to complete the desired movement without the interfering tensions (Alexander and Maisel, 1989). This is a clear example of working with the PS; Jones, a student of Alexander's published a book summarizing his early investigations of the AT as producing change through the inhibition of postural sets (Jones, 1976). Jones makes it clear that the “postural set” is not the simple adoption of a physical posture, but an integrated change in attitude involving attentional, cognitive and affective components, largely equivalent to our concept of the PS. The “inhibition” referred to by Jones and Alexander is the process of relinquishing chronic maladaptive sets, very similar to the process we discuss above under Changing PS. Jones documents the increased mechanical efficiency of movement (using stroboscopic photography) as well as the subjective experiences of lightness, height and smoothness (in a questionnaire given to new students) (Jones, 1976). Extensive anecdotal and informal written accounts, as well as one of the authors' clinical experience, suggests that students also experience positive changes in physical and emotional health and improved cognitive function, consistent with our view that the PS links motor, affective and cognitive functioning.

There have been few scientific studies of the AT. It has been shown to be more effective than massage in treating back pain (Little et al., 2008), to increase functional reach (Dennis, 1999), to benefit patients with Parkinson's disease (Stallibrass et al., 2002), asthma (Dennis and Cates, 2000), and performance anxiety (Urbanski, 2012); for general reviews, see (Ernst and Canter, 2004; Jain et al., 2004). Stuart offers an interesting discussion of the AT and neurophenomenology (Stuart, 2013).

##### Feldenkrais Method

Strongly influenced by the AT, the Feldenkrais Method (FM) uses either complex movements guided by verbal instruction (Awareness through Movement) (Feldenkrais, 1990) or passive manipulation of the body by an instructor (Functional Integration) (Rywerant and Feldenkrais, 2003). It emphasizes proprioceptive awareness, fine motor discrimination, and the use of imagined movement. The stated aim is to offer the motor nervous systems a wider range of alternatives for movement, removing the constraints of limited habit patterns; the assumption is that the nervous system will then automatically choose the most efficient alternatives. Feldenkrais believes these changes will positively affect emotional and cognitive state; his theories are congruent with our hypothesis of the PS. Extensive anecdotal evidence supports this claim although there are only a few published studies. Most studies have positive results (Gutman et al., 1977; James et al., 1998; Lundblad et al., 1999; Kolt and McConville, 2000; Gomes and Vieira, 2013; Ramli et al., 2013; Pugh and Williams, 2014), although they are generally of poor quality.

A study of the use of language in the teaching of physical education versus the FM provides an interesting perspective on the differing world-views of BTES and conventional physical education (Wright, 2000).

##### Rolfing

Rolfing adopts a point of view similar to that of AT and FM, that physical structure and movement are foundational to well-being and have intrinsic links to autonomic, affective, cognitive and attentional aspects of the person (Rolf, 1977). This is congruent with our hypothesis. Rolfing uses deep massage on the connective tissue to undo the effects of chronic patterns of poor posture and movement (Larson, 1990). In an extensive but non-scientific literature, participants report substantial alterations in proprioceptive and interoceptive experience that affect their emotional and mental state, including transformative emotional releases and spontaneous re-evaluations of past trauma (Fahey, 1989; Karrasch, 2009). We suggest that the altered proprioceptive feedback from the muscles, and the newly available freedom of movement, may alter PSs and allow the subject to have a different “attitude” toward life; an attitude with affective and cognitive repercussions. This hypothesis is similar to the stated theory of Rolfing (Fahey, 1989; Sise, 2005).

There has been little scientific research on Rolfing. Studies are of poor quality and point to the need for more research and for the development of appropriate outcome measures. Studies have shown positive effects on balance (Findley et al., 2004), parasympathetic tone and pelvic angle (Cottingham et al., 1988) and neck pain (James et al., 2009), but no effect was shown on cerebral palsy based on measures of limb range of movement and gait velocity and efficiency (Perry et al., 1981).

#### Body psychotherapy

Body-oriented psychotherapy pre-dates the development of verbal psychotherapies. Freud's mentor Pierre Janet first formulated and practiced body-oriented psychotherapy, using massage, breathing techniques, and guided movement to encourage the release of emotional blockages (Boadella, 1859). Freud initially used these techniques, but later abandoned them to focus on cognitive insight and the working through of the relationship between therapist and patient. Freud's student Reich continued to develop body-oriented psychotherapy (Boadella, 1974), and strongly influenced later methods (Boadella, 1976).

There has been little scientific research on body psychotherapy. There have been studies (more in Europe than in the States) which confirm its effectiveness for severe mental disorders (Ventling, 2002; Price et al., 2007, 2012; Levy Berg et al., 2009; Röhricht, 2014). Surprisingly, there has been little investigation into its mechanisms, although current neuropsychology seems to offer supportive and suggestive evidence. For a good survey of the field see (Heller, 2012). Exposure therapy may share some of the mechanisms of body psychotherapy, although the theory behind it is quite different (McNally, 2007).

##### Reichian Therapy

Reichian Therapy maintains that dysfunctional emotional/cognitive patterns are embodied in patterns of muscular tension (“character armor”) (Boadella, 1974). In addition to insight techniques, Reich used massage, breathing exercises and guided movement to release the tensions. He believed that the tension patterns served to hold back instinctive emotional, physical and sexual impulses, blocking the natural charge/discharge process of the body (Boadella, 1974). The holding patterns identified by Reich are very similar to our concept of maladaptive persistent PSs: the restraint of instinctive responses may lead to a failure of the PS to subside and its persistence as a maladaptive pattern. Note that in our discussion of types of PS above, we suggest that *any* of the core emotional patterns (described by Panksepp) may lead to maladaptive PSs, including sexual responses and nurture-oriented impulses as well as the more familiar fear and rage responses. Reich's idea of health as a flexible state in which impulses are not chronically suppressed but allowed healthy expression is congruent with our suggestion that stress can be alleviated by the release of persistent and disorganized PSs.

In his later years Reich brought elements into his approach which eventually drew legal action from the government; his books were burned and he ended his life in prison. His successors have avoided these extremes, and have developed the embodied aspects of his approach, discarding the more analytical Freudian legacy.

##### Formative Psychology

Stanley Keleman's Formative Psychology (Keleman, 1979) is a sophisticated refinement of Reich's approach. Keleman emphasizes the use of conscious voluntary effort in forming ones attitude toward life and life events. “Forming” is not a metaphor, but literally the adopting of a certain shape; flexing or extending, shrinking or inflating, altering both musculo-skeletal configuration as well as visceral tone. According to Keleman, problems arise when, through early childhood events, we get “stuck” in a certain shape (Keleman, 1971); we adopt a fixed, maladaptive attitude toward life, which may be automatic and unconscious, inaccessible for modification. Keleman draws the attention of his clients to “how they do that,” to the proprioceptive, interoceptive and kinesthetic subtle cues through which they can recover awareness of the original action, and make a voluntary choice to un-do it and to form oneself in a more functional way (Keleman, 2013). His approach is thus very similar to our hypothesis of the PS.

##### Somatic Experiencing

Peter A. Levine, is a PhD in psychology and psychophysiology, trained as a body psychotherapist and as a Rolfer. He developed Somatic Experiencing, a form of psycho-physical therapy specifically designed to resolve the effects of traumatic stress. He noted that animals in the wild, subject to extremes of stress, may exhibit freeze or collapse behavior similar to that of humans with PTSD (Hoagland, 1928; Nijenhuis et al., 1998). This has been documented as TI (Volchan et al., 2011). In animals this state seemed to generally be time-limited; the animal would go through an apparent physical “discharge” process, involving heavy breathing, shaking or trembling, and subtle locomotor motions; after which they appeared to have thrown off the effects of the stress (Levine and Buczynski, 2013). These naturalistic observations have not yet been documented in the literature but have been recorded on film by wildlife biologists (Lipscomb, 1982). SE addresses PTS by guiding the client to become aware, through interoception and proprioception, of unresolved defensive impulses, and then releasing these impulses through biological completion, described above.

The theories of SE are congruent with the PS framework, and the PS framework provides an explanation for the potential effectiveness of SE. Its methods and theory are discussed in Payne et al. (2015). Peer-reviewed research publications into the efficacy of SE methods are as yet limited to special applications of SE (See Payne et al., 2015–Corrigendum). Its methods and theory are discussed in Payne et al. (2015).

### Suggestions for further research

Our hypothesis is that the five aspects of the PS–posture, autonomic state, affective state, attention, and expectation—tend to co-vary, suggesting a central integrated response we term the PS. Further, that the BTES involve changing one or more of these with the aim of changing the others too, bringing about a re-organization of PS, a more adaptive state of the organism, and a reduction of physiological and psychological markers of excess ergotropic activity (the most common usage of the term “stress”).

There are readily available means for measuring the five aspects of the PS:

- Autonomic state: heart rate, breath rate, blood pressure, heart rate variability, galvanic skin response, as well as a number of biochemical markers such as C-reactive protein, catecholamine and steroid hormone levels.
- Affective state: any of a number of questionnaires. Both autonomic and affective reactivity may be tested using picture-viewing procedures accompanied by autonomic and affective measures (Lang et al., 1993).
- Expectation may be tested in several ways, including subliminal word perception threshold (Marcel, 1983).
- Attention could be tested with breadth-of-field experiments, gaze direction monitoring, flicker-fusion and word- or image-recognition threshold tests; such tests have already been used in evaluating the effects of meditation (Brown et al., 1984).
- Objective measurements of the kind of postural changes relevant to this context could include strobe photography (to measure the efficiency of simple movement patterns) (Jones, 1976), electromyography (to measure tension in selected muscle groups), and the use of moiré patterns to measure subtle changes in postural alignment (Meadows et al., 1970; Gertzbein et al., 1985).

A randomized controlled study could evaluate BTES interventions on one of these variables by using the other variables as outcome measures. We would suggest the use of an attention control group as well as an active control using a standard intervention such as relaxation or Cognitive therapy.

For example, one could monitor autonomic reactivity to a challenge such as picture viewing (Lang et al., 1993), and determine whether the guidance by a teacher of Qigong or the Alexander technique into a “centered” postures would bring about an alteration in reactivity, in comparison to a control activity or to a self-adopted posture. Further, one could determine whether this same postural guidance also brought about a widening of peripheral attention, a more positive set of expectations, and a more positive affective state, as our hypothesis would suggest. Similar experiments could be done with a BTES intervention on autonomic state (using controlled breathing), affective state (using methods from body psychotherapy), attention, or expectation.

Our hypothesis that the neural substructures involved in the PS are largely sub-cortical is more challenging to verify in humans, as it is technically demanding to monitor subcortical activity in humans since it is situated deep in the brain. Panksepp makes a case for the validity of generalizing from animal data to humans (Panksepp, 2011). fMRI studies of humans undergoing BTES procedures are possible in some cases, although hampered by the limitations the fMRI procedure places on movement. Pre- post-procedure fMRIs would yield useful data on subcortical changes following BTES interventions.

As noted in several places above, experiments of this sort have already been done with results suggesting support of our hypotheses.

## Summary

Here we have proposed that the concept of the PS as an important but underemphasized phase of the organism's response to challenge. The PS is an integrated, largely sub-cortical, organization of the organism in readiness to handle events. We suggest that the PS may persist inappropriately or become disorganized in states of chronic or traumatic stress, and that intervention through attention to interoceptive and proprioceptive experience is more likely to be an effective therapeutic approach than one focused on verbal meaning. We have laid out the various aspects of the PS—movement, posture, orientation, autonomic state, emotion, expectation—and the neurological substrates of each. We suggest that since the PS is primarily subcortical, it is quicker and more fundamental, and may override or bias cortical executive function. We have presented a brief overview of various forms of BTES, and suggest that they share an approach very similar to the one we present with the PS framework, and that the PS framework therefore offers a way of approaching the neuroscience of the BTES. We hope that our suggestions facilitate further research and integration of these concepts that have application into virtually all aspects of human health and well-being.

### Conflict of interest statement

Peter Payne is an SE practitioner (SEP) who derives income from his practice. Mardi A. Crane-Godreau is an SEP and a non-paid member of the Board of Directors of the Somatic Experiencing Trauma Institute.
